# Manuka honey treatment of biofilms of *Pseudomonas aeruginosa* results in the emergence of isolates with increased honey resistance

**DOI:** 10.1186/1476-0711-13-19

**Published:** 2014-05-12

**Authors:** Aimee L Camplin, Sarah E Maddocks

**Affiliations:** 1 Cardiff Metropolitan University, Western Avenue, Cardiff CF5 2YB, UK

**Keywords:** Antimicrobial, Small colony variant

## Abstract

**Background:**

Medical grade manuka honeys are well known to be efficacious against *Pseudomonas aeruginosa* being bactericidal and inhibiting the development of biofilms; moreover manuka honey effectively kills *P. aeruginosa* embedded within an established biofilm. Sustained honey resistance has not been previously documented for planktonic or biofilm *P. aeruginosa*.

**Methods:**

Minimum inhibitory concentrations for manuka honey and antibiotics were determined using broth micro-dilution methods. Minimum biofilm eliminating concentrations (MBEC) and biofilm biomass were determined using the crystal violet method. Sub-culture used non-selective media and the grid-plate method.

**Results:**

When honey treated biofilm biomass of two strains of *P. aeruginosa* (reference strain ATCC 9027 and the clinical isolate 867) were sub-cultured onto non-selective media isolates emerged that exhibited reduced susceptibility to manuka honey. Significantly, this characteristic was sustained with repeated sub-culture onto non-selective media resulting in increased minimum inhibitory concentrations (MIC) of between 5-7% (w/v) and increased minimum biofilm eliminating concentrations (MBEC) of up to 15% (w/v). Interestingly the resistant isolates showed reduced susceptibility to antibiotic treatment with rifampicin and imipenem as well as being more prolific biofilm-formers than the progenitor strains.

**Conclusions:**

*P. aeruginosa* biofilms treated with manuka honey equivalent to the MBEC harbour slow growing, viable persistor organisms that exhibit sustained, increased resistance to manuka honey and antibiotic treatment, suggesting a shared mechanism of resistance. This sheds new light on the propensity for biofilm embedded organisms to resist honey treatment and become persistor organisms that are tolerant to other antimicrobial therapies.

## Background

Honey has been long utilised as a topical antimicrobial treatment for wound infection, with licensed medical products becoming available in the 1990’s. Whilst often still used as a last line treatment when other therapies have failed, manuka honey is broad spectrum with potent bactericidal activity. Some of the bacterial cellular targets for manuka honey are now known and more continue to be identified. For Gram positive organisms the primary mode of action is to disrupt the cell cycle leading to aberrant cell division and weakening of the cell wall; this is combined with an up-regulation of the general stress response [1,2]. In Gram negative organisms, particularly *Pseudomonas aeruginosa*, the major targets appear to be integral membrane proteins such as OprF which ordinarily stabilise the cell leaflet and without which, extensive membrane disruption and eventual lysis occurs [3].

Essentially, studies have demonstrated that with short- and long-term training experiments, no resistance to manuka honey treatment emerges [4,5]. This is of particular significance in an age where antimicrobial resistance outstrips the rate at which new antimicrobial treatments are discovered and where there is real concern that species resistant to all known antibiotics could become rife. The problem of resistance can be exacerbated, for example when microorganisms grow as a biofilm. Such organisms are afforded intrinsic protection in the form of an extra-polysaccharide layer which restricts the diffusion of antimicrobials meaning that therapeutic doses do not reach all bacteria within the biofilm, causing infection to recur [6,7]. Therefore the biofilm mode of growth, by hindering diffusion, could provide an ideal environment whereby microorganisms are exposed to sub-lethal doses of antimicrobial thus providing a selective pressure for the emergence of resistance.

Previous studies have shown that following treatment of established biofilms of *P. aeruginosa* biofilms, the bacterial biomass appears non-viable by fluorescent microscopy, but with a large amount remaining attached to the sub-stratum [8]. This study aimed to determine whether viable cells were in fact present within this biomass, since the published data showed only top-views of biofilms [8] rather than three-dimensional Z-stacks that might have revealed any embedded viable bacteria, deeper within the biofilm, and if so to ascertain whether these persistors exhibited resistance to manuka honey treatment. Here we report that isolates of *P. aeruginosa,* recovered from manuka honey treated biofilms, which appeared to contain only non-viable biomass, exhibited increased resistance to manuka honey treatment, a trait that was sustained despite repeated sub-culture. These isolates were also more resistant to other antibiotics and demonstrated enhanced biofilm forming capacity.

## Methods

### Bacterial strains

*P. aeruginosa* reference strain ATCC 9027 (NCIMB 8626) and the clinical isolate 867 were used throughout the study. The clinical isolate was from a wound swab collected from a patient with a chronic wound who attended an out-patient clinic at the University Hospital of Wales, Cardiff. All strains were cultured aerobically at 37°C in nutrient broth (NB). Biofilms were cultured in 5 ml NB supplemented with manuka honey at the appropriate MBEC (50% w/v for ATCC 9207 and 45% w/v for 867) in a sterile plastic Petri dish for 48 h at 37°C, biomass was recovered by scraping the plate into 1 ml PBS, re-suspending by vortex and lawn plating onto nutrient agar (NA), these were incubated at 37°C for 48 h. Isolates were confirmed as *P. aeruginosa* by Gram stain, oxidase test and production of blue/green pigment. Isolates were sub-cultured every 48 h for two weeks onto non-selective media to confirm that any observed resistance was a sustained and not transient.

### Manuka honey

Sterile (gamma irradiated) medical grade manuka honey (Medihoney™) was purchased from Comvita in 50 g tubes, and is available as a licensed, commercial medical device (Comvita, Berkshire, UK). It is supplied as a standardised, 100% pure honey derived from the *Leptospermum scoparium* plant in New Zealand.

### Minimum inhibitory concentration

Minimum inhibitory concentrations (MIC) for manuka honey were determined using a range of concentrations of 0-50% (w/v), in increments of 2%), in a total volume of 50 μl nutrient broth in a 96 well microtitre plate (Oxoid, Cambridge, UK). Plates were incubated for 48 h to allow for slow growth of the recovered isolates, at 37°C in aerobic conditions. Assays were carried out in triplicate on each of three separate occasions. MIC readings were taken using a Spectrostar Nano spectrophotometer (BMG Labtech) at *A*_650_. MICs for imipenem and rifampicin were determined by preparing doubling dilutions ranging from 0-64 μg ml^-1^ in a total volume of 50 μl NB. These antibiotics were chosen due to their known efficacy against the two strains of *P. aeruginosa* used in this study [9,10]. Plates were incubated and read as described above.

### Minimum biofilm eliminating concentration

Microorganisms were cultured to mid-exponential phase, harvested by centrifugation, and adjusted to OD_650_ = 0.1. Biofilms were established in 96 well microtitre plates (Greiner) in 50 μl NB containing a range of concentrations of manuka honey from 0-80% (w/v; increments of 5%), by inoculating each well with 5 μl of equilibrated cells. Plates were incubated at 37°C for 48 h. To determine biofilm growth, unattached cells were gently aspirated and discarded, adherent cells were washed twice with PBS and then stained with crystal violet (0.25% w/v) for 10 min. After a subsequent two washes with PBS, cell-bound crystal violet was re-solubilized with 7% acetic acid, and absorbance measured at *A*_595_[11].

### Biofilm biomass assay

Microorganisms were cultured to mid-exponential phase, harvested by centrifugation, and adjusted to OD_650_ = 0.1. Biofilms of recovered isolates were established in 96 well microtitre plates (Greiner) in 50 μl NB, by inoculating each well with 5 μl of equilibrated cells. Plates were incubated at 37°C for 48 h. Biomass was determined as described above.

### Statistical analysis

Triplicate sets of biological replicates were used for each experiment and each assay was repeated three times; statistical analysis utilized Minitab (version 14; Students *T*-test). Statistically significance data are defined as having P values that were equal to, or less than 0.05.

## Results

### Isolates recovered from biofilm biomass showed sustained, increased resistance to manuka honey treatment when cultured planktonically and as a biofilm

The MIC for the reference strain ATCC 9027 was lower than the recovered isolate, being 25.6% (w/v) and 30.6% (w/v), respectively (*p* < 0.05) (Table 1); similarly the clinical isolate, strain 867 showed an increase in MIC from 15.3% (w/v) for the progenitor to 22.6% (w/v) for the recovered isolate (*p* < 0.05). Likewise, each recovered isolate had higher MBEC values than the progenitor strain, for ATCC 9027 MBEC increased from 48.3% (w/v) to 63.3% (w/v) (*p* < 0.05) and for the clinical isolate 867 the MBEC increased from 43.3% (w/v) to 50% (w/v) (*p* < 0.05). Following repeated sub-culture the MIC/MBECs remained consistent and did not revert to those of the progenitor strains, suggesting that the recovered isolates had developed increased resistance to manuka honey treatment.

**Table 1:** **Sensitivity of progenitor strains *P. aeruginosa *** **ATCC 9027 and clinical isolate 867 and their recovered isolates (from honey treated biofilm) to manuka honey treatment using the microbroth dilution method**

	**Mean MIC% (w/v) ± SE**	
	***P. aeruginosa *** **ATCC 9027**	***P. aeruginosa *** **867**
Progenitor strain	25.6 ± 0.6	15.3 ± 0.6
Recovered isolate	30.6 ± 0.6	22.6 ± 0.6
***p-*** **value**	**<0.05**	**<0.05**

SE = standard error of the mean.

### Honey resistant isolates demonstrated increased resistance to treatment with rifampicin and imipenem

To determine whether the observed increase in tolerance to manuka honey treatment also conferred increased resistance to antibiotics, MICs for each of the progenitor and recovered isolates using rifampicin and imipenem were established. Both recovered isolates exhibited increased resistance to imipenem and rifampicin (Table 2) with MICs at least doubling in each case. For ATCC 9027 the MIC for imipenem increased from 2.6 μg ml^-1^ for the progenitor to 13.3 μg ml^-1^ for the recovered isolate (*p* < 0.05); with rifampicin the MIC increased from 13.3 μg ml^-1^ to 32 μg ml^-1^ (*p* < 0.05) (Table 2). For strain 867 the MIC for imipenem increased from 8 μg ml^-1^ for the progenitor to 21.3 μg ml^-1^ (*p* < 0.05) for the recovered isolate; for rifampicin the MIC increased from 13.3 μg ml^-1^ for the progenitor to 32 μg ml^-1^ (*p* < 0.05) for the recovered isolate (Table 2).

**Table 2:** **Sensitivity of progenitor strains *P. aeruginosa *** **ATCC 9027 and clinical isolate 867 and their recovered isolates (from honey treated biofilm) to rifampicin and imipenem treatment using the microbroth dilution method**

	**Mean MIC μg ml**^**-1**^ **± SE**			
	***P. aeruginosa *** **ATCC 9027**		***P. aeruginosa *** **867**	
	**Rifampicin**	**Imipenem**	**Rifampicin**	**Imipenem**
Progenitor strain	13.3 ± 2.6	2.6 ± 0.6	8 ± 0	13.3 ± 2.6
Recovered isolate	32 ± 0	13.3 ± 2.6	21.3 ± 5.3	32 ± 0
*p-*value	<0.05	<0.05	<0.05	<0.05

SE = standard error of the mean.

### Honey resistant isolates exhibited increased biofilm forming capacity

When static biofilms of each recovered isolate were cultured for 48 h and stained with crystal violet, the biomass appeared markedly increased compared to that of the progenitor strains cultured under the same conditions (Table 3). For strain ATCC 9207 a 3.8 fold increase in biomass was observed for the recovered isolate compared to the progenitor strain, and for the clinical isolate 867 an increase of 2.1 fold was evident. This suggests that the recovered isolates have a better capacity for forming biofilm, despite being slower growing than the progenitor strains.

**Table 3:** **Increase in biofilm biomass of progenitor strains *P. aeruginosa *** **ATCC 9027 and clinical isolate 867 and their recovered isolates (from honey treated biofilm) using the crystal violet method**

	**Biofilm biomass (A**_**595**_**) ± SE**	
	***P. aeruginosa *** **ATCC 9027**	***P. aeruginosa *** **867**
Progenitor strain	0.15 ± 0.01	0.18 ± 0.02
Recovered isolate	0.58 ± 0.1	0.39 ± 0.02
***p-*** **value**	**<0.05**	**<0.05**
**Fold-increase**	**3.86**	**2.16**

SE = standard error of the mean.

## Discussion

Excessive and inappropriate use of antimicrobial agents has long been acknowledged as a driver for the emergence of resistant strains of bacteria, and there is a correlation between the availability of an antimicrobial and occurrence of resistance within a microbial population [12-15]. This is not limited to antibiotics, resistance to topical antiseptics and biocides that are often used to treat wound infection, including chlorhexidine and silver, have been comprehensively documented [16-19]. Bacteria acclimatise quickly to antimicrobials; it can be a matter of months between the introduction of a new antimicrobial and the appearance of resistant strains. However, when the selective pressure is removed, by restricted use, a reduction in the occurrence of resistant isolates is often observed [5]. Short and long term, step-wise resistance training has previously demonstrated that neither *P. aeruginosa* nor *S. aureus* develop resistance to honey and that any increased tolerance to treatment is transient [4,5]. In these studies planktonic cultures were repeatedly sub-cultured in the presence of sub-inhibitory doses of honey with MIC values obtained daily with each transfer.

Our study showed that viable, slow growing isolates that were recovered from the biomass of biofilms treated with honey and which appeared to be non-viable by fluorescent microscopy [8], had increased resistance to manuka honey treatment. Moreover this was statistically significant (*p* < 0.05) with increased MICs for isolates grown planktonically and also increased MBECs (Tables 1 and 4). Repeated sub-culture of the recovered isolates did not result in reversion to the more susceptible, progenitor phenotype for either strain (ATCC 9027 and clinical isolate 867). Within the biofilm diffusion of antimicrobial agents can be impaired by the polysaccharide matrix, resulting in a failure to reach inhibitory or bactericidal concentrations throughout the whole biofilm [6,7]. Therefore it is likely that these sub-inhibitory/sub-lethal levels instead provide a selective pressure that favours sustained resistance.

**Table 4:** **Sensitivity of biofilms of progenitor strains *P. aeruginosa *** **ATCC 9027 and clinical isolate 867 and their recovered isolates (from honey treated biofilm) to manuka honey treatment using the crystal violet method**

	**Mean MBEC% (w/v) ± SE**	
	***P. aeruginosa *** **ATCC 9027**	***P. aeruginosa *** **867**
Progenitor strain	48.3 ± 1.6	43.3 ± 1.6
Recovered isolate	63.3 ± 1.6	50 ± 0
*p-*value	<0.05	<0.05

SE = standard error of the mean.

It was not possible to investigate possible mechanisms of resistance within the remit of this study but it was interesting to note that the honey resistant recovered isolates of each strain also exhibited increased resistance to rifampicin and imipenem (Table 2). It is possible therefore that mutations have occurred in these strains that confer cross-resistance to these antimicrobials, but given the different modes of action of these antimicrobials it could be hypothesised that the recovered isolates, which were slower growing than the progenitor strains, were small colony variants (SCV) of the original isolates. SCVs can emerge in populations of bacteria that are exposed to stresses which results in transient or permanent phenotypic changes, typically including increased resistance to antimicrobial treatment [19]. They can occur at a rate of 1.8×10^-8^ per cell per generation, comprising a small but persistent proportion of the bacterial population [20]. The propensity for SCVs to resist antimicrobial treatment is attributed to their slow growth and impaired electron transport chain; therefore it is not uncommon for SCVs to show increased resistance to several different classes of antimicrobial [21], which might be the case in this study. Furthermore SCVs characteristically show enhanced biofilm formation [22] which was observed here and might also explain why the MBEC for manuka honey was increased compared to biofilm of the progenitor strains.

The European Committee on Antimicrobial Susceptibility Testing (EUCAST) defines antimicrobial resistant microorganisms as being resistant by a level of antimicrobial activity that is likely to result in therapeutic failure [23]. Licensed medical products containing manuka honey do so at levels of between 80-95% (w/v) [5] which exceeds the *in vitro* MICs and MBECs observed in this study. However wounds often produce exudate and the high osmolarity of honey promotes this meaning that honey is invariably diluted *in situ*. Since this is highly dependent on the nature of the wound the actual magnitude of dilution is not known and could have the potential to exacerbate the likelihood of resistance, especially in infections where biofilm is present. Despite this study indicating that increased resistance to manuka honey was concurrent with increased resistance to imipenem and rifampicin, combined use of these antimicrobials with honey could outweigh problems that might be associated with resistance as they have been shown to act synergistically to increase microbial susceptibility *in vitro*[9,10]; however such studies did not use biofilms. The authors are currently investigating the combined effects of honey and antibiotics on our resistant isolates to ascertain whether they are susceptible to combination therapy.

## Conclusions

In conclusion, our studies reveal for the first time that isolates exposed to manuka honey within an established biofilm can develop sustained, increased resistance, which is possibly due to the appearance of small colony variants within the microbial population. This is a factor that needs to be taken into consideration in clinical practice where both biofilm organisms and small colony variants are often overlooked due to difficulties isolating them by standard techniques *in vitro*. Therefore where recalcitrant or chronic, infected wounds are present it remains vital to ensure that topical treatments such as manuka honey are appropriately applied for a suitable length of time in combination with other antimicrobials where necessary to ensure that infection is resolved and the likelihood for resistance is minimised.

## Abbreviations

SCV: Amall colony variant; MIC: Minimum inhibitory concentration; MBEC: Minimum biofilm exclusion concentration.

## Competing interests

The authors declare that they have no competing interests.

## Authors’ contributions

AC carried out all of the experiments described in this paper, including statistical analysis. SM conceived of the idea and designed the experimental work. Both authors read and approved the final manuscript.
